# Physiology Vindicated, in a Critique on Liebig’s Animal Chemistry

**Published:** 1843-09-16

**Authors:** 


*Physiology Vindicated, in a Critique on Liebig's Animal Chemistry.* By Charles Caldwell, M. D., Jeffersonville, Ia. 1843. 8vo. pp. 95.

The advance of Animal Chemistry within the past few years, and its connection with Physiology, has alarmed Dr. Caldwell, and as one of the "sentinels" of science, he considered it incumbent upon himself, not only to announce the approach of danger, but also to assume the character of a "guardian," "and even of a combatant, and protect from unwarrantable inroads the branch of science committed to his superintendence," (Preface p. v.) In this threefold character "of three gentlemen at once," Dr. Caldwell appears before us to oppose the "ambition and rapaciousnsss," as well as the "aggressions," supposed or real, of Organic Chemistry, which under the command of its Chief, Professor Liebig, indicates, he thinks, "a determination to usurp dominion over the whole philosophy of living organized matter." With all the ability of an old and skilful tactitian and accomplished warrior, Professor Caldwell aims a decisive blow at the leader of this predatory band, whom, he thinks, "without any inordinate extravagance of hyperbole" (!) may be likened to Attila, the "Curse of God," "beneath the hoofs of whose war [hobby?] horse" the verdure of the physiological parterre committed to the care of the Louisville Professor is "blighted." "The interests of the *philosophy of Medicine,*" which our author has been "deputed to teach, being thus endangered by the celebrated German Professor and his followers," he thought that it did not comport with his "sense of either moral or official duty, to remain longer inactive.' The doctrine which "metamorphoses man with all his attributes, corporeal and mental, into a chemical product," Dr. Caldwell considers as "not only physically groundless, but morally pernicious." From this chemical creed thus "groundless, degrading, and pernicious," and menacing religion, morality and philosophy, has arisen the "unspeakable mischiefs and miseries of humoralism."

These are themes for thought; but we touch them not. We have thus given the scope and tendency of this publication, which we have perused with patience, and closed with a distressing impression of the amount of labour the author has bestowed on his composition. He tells us (Preface, p. vi.) that finding the character of a controversialist unprofitable and thankless from a long experience, he "sickened at the prospect of a new collision;" but a stern sense of duty overcame all inferior considerations, and he boldly buckled on his armour, somewhat the worse for wear, and launched into the thickest of the combat. He regrets his insufficient preparation for the attack, but relying, doubtless, on his name and well-tried abilities, he sallies forth; but like other whilome veterans, with inadequate notions of the nature of the territory he is about to invade, an erroneous idea of the strength of the enemy, in utter ignorance of the system of tactics they employ, and with a pretty certain prospect of defeat, should any of the tyros of the opposite cause have leisure for a little pastime.

In the whole of the ninety-five pages which it was our misfortune to wade through, we have not found a single plausible argument, or a solitary fact opposed to the reasoning or the statements of Liebig. If the Professor of Gissen has erred in theory, or been premature in many of his conclusions, not a single proof of his faults could be adduced from the pedantic slip-shop of this pamphlet. When a man does a dull thing in a quiet way, we are inclined to pardon or overlook it; but we consider the inconcievable stupidity that gratuitously thrusts forth its crude, turgid and operose ignorance, in an emphatic and arrogant tone, as worthy of castigation, as of the irresistible ridicule it invariably excites. On this occasion we do not know if we were more provoked at the conceit, bad taste, and perversity of the manifold affectations of the author, pained by the perpetual tone of toilsome effort, and rhetorical exaggeration pervading the book, or startled at the wretched twaddle which fills page after page.

